# Is OpenSDE an alternative for dedicated medical research databases? An example in coronary surgery

**DOI:** 10.1186/1472-6947-7-31

**Published:** 2007-10-22

**Authors:** Angeliek C Venema, Astrid M van Ginneken, Marcel de Wilde, Ad JJC Bogers

**Affiliations:** 1 Dept. of Cardio-Thoracic Surgery, Erasmus MC, Rotterdam, The Netherlands; 2 Dept. of Medical Informatics, Erasmus MC, Rotterdam, The Netherlands

## Abstract

**Background:**

When using a conventional relational database approach to collect and query data in the context of specific clinical studies, a study with a new data set usually requires the design of a new database and entry forms. OpenSDE (SDE = Structured Data Entry) is intended to provide a flexible and intuitive way to create databases and entry forms for the collection of data in a structured format.

This study illustrates the use of OpenSDE as a potential alternative to a conventional approach with respect to data modelling, database creation, data entry, and data extraction.

**Methods:**

A database and entry forms are created using OpenSDE and MSAccess to support collection of coronary surgery data, based on the Adult Cardiac Surgery Data Set of the Society of Thoracic Surgeons. Data of 52 cases are entered and nine different queries are designed, and executed on both databases.

**Results:**

Design of the data model and the creation of entry forms were experienced as more intuitive and less labor intensive with OpenSDE. Both resulting databases provided sufficient expressiveness to accommodate the data set. Data entry was more flexible with OpenSDE. Queries produced equal and correct results with comparable effort.

**Conclusion:**

For prospective studies involving well-defined and straight forward data sets, OpenSDE deserves to be considered as an alternative to the conventional approach.

## Background

Acquisition of patient data for clinical research is challenging, because routinely collected patient data is often incomplete, fragmented (divided over different data sources), or poorly accessible (on paper or in free text format). Therefore, clinical research projects usually involve dedicated data collection in addition to data recording for routine care. It is quite common for researchers to develop a new dedicated database with data entry screens, each time a new data set is required. Is there an alternative approach for the relatively labor-intensive development of dedicated research databases and separate data collection effort?

OpenSDE (SDE = Structured Data Entry) has been developed to support structured recording of data for both research and care, and is designed to accommodate quickly growing and changing data sets [1]. An essential characteristic of OpenSDE is the separation of data content from database structure. OpenSDE, based on a row-oriented data model, offers flexible and intuitive definition and adaptation of content without the need to change the underlying data model or user-interface. Because of the high expressiveness of OpenSDE and the customizability of forms for data entry and consultation, our academic hospital has adopted OpenSDE functionality in the electronic patient record (Elpado). OpenSDE functionality is currently used in routine clinical practice in the departments of sexually transmitted disease, gynecology, and neurology. Ten other departments are in an advanced stage of record development with OpenSDE functionality.

OpenSDE is also used for several specific clinical research projects (e.g. Tall stature study, disorders of sexual development, CT for Head Injury Patients study, factors influencing growth in children). Main reason for using OpenSDE, both in the clinical and the research settings, is the flexible and intuitive way of creating a database and user interface for data entry [1-5].

Over the past years, we have often been confronted with questions regarding extraction of data collected with OpenSDE, but so far, only one of our publications focus on extraction of routinely collected patient data [6]. When the Cardio-Thoracic surgeons were introduced to OpenSDE in the context of computerized patient records, they wondered if this application would be useful for specific well-defined clinical studies. Therefore, we decided to apply OpenSDE to the domain of coronary surgery and explore the use of OpenSDE as a potential alternative to the conventional dedicated approach.

To illustrate the use of OpenSDE as a potential alternative to a conventional relational approach, we used both approaches to collect and extract specific data in the domain of coronary surgery. All steps in the process pass in review: data modelling, database creation, data entry and data extraction.

Since data modelling and data entry with OpenSDE have extensively been described elsewhere, emphasis is on data extraction.

## Methods

### Materials

#### OpenSDE

OpenSDE uses a row-oriented database to achieve flexibility in content coverage. Row-modelling involves a column-to-row transformation, where the new columns are generic. This strategy implies a higher level of abstraction, such that changes to content coverage do not require adaptations to the database structure [7].

The fact that the row-oriented data model itself is abstract means that semantics (meaning and context of the data) are not represented by the database structure [8]. In the model used by OpenSDE, context is not represented through internal row reference (within the data), but through references to separately defined metadata in the form of domain-specific trees [9].

A tree represents, for a specific domain, which entities can be described and in what context. A node in the tree is a medical concept and its branching nodes represent its descriptors. An essential principle while constructing a tree is that a concept is represented once, i.e. as a unique node, in the tree. The tree has no 'knowledge' of what nodes are conceptually the same. This is because a path from top to node represents a concept, not the node itself. Hence 'severity' of 'cough' differs from 'severity' of 'chest pain'. Besides the ordering of nodes in a tree, each node has several properties, which define options and constraints for data entry (e.g. plausible min. and max. for a value). Custom constraints can also be defined (e.g. systolic blood pressure must be higher than the diastolic blood pressure).

Trees are created and maintained via an interactive editor, the domain model editor. Besides basic data types, such as categorical values, numeric, text, and temporal, the expressiveness of OpenSDE also encompasses ranges of values, multiple occurrence of the same tree node (two different skin lesions), progress descriptions (multiple descriptions of the same node to represent course over time), comments, and time-stamps.

OpenSDE automatically generates standard entry forms, based on the contents of the tree. For each node, the standard entry form contains entry options for its descriptors. Users can also define custom forms with self-defined sets of tree-nodes to suit specific medical contexts or tasks [10]. These custom forms accommodate the entry of concepts that are relevant in multiple medical contexts.

During data entry, the user can traverse the tree to select concepts for description. Upon selection of a concept, OpenSDE presents the associated standard or custom form for data entry.

**Figure 1:**
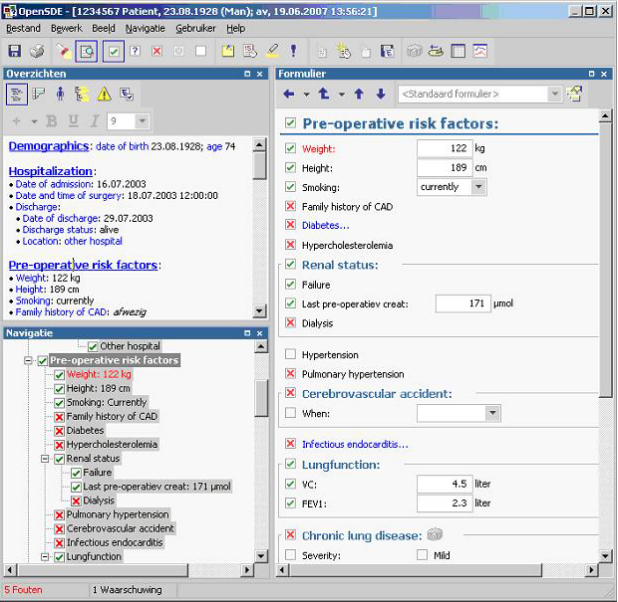
**Screen capture of the OpenSDE user-interface**. Example of the OpenSDE user interface. At the top left is an overview of the data entered so far in the current session. The bottom left shows the domain tree. The entry form on the right is associated with the selected concept in the tree (Pre-operative riskfactors).

Figure 1 shows an example of the OpenSDE user-interface.

**Figure 2:**
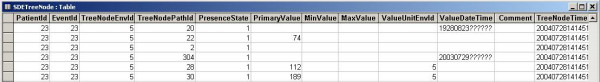
**Screen capture of the table SDETreeNode in OpenSDE**. The first row contains the column headers. The first three columns represent references to the patient, event (registration session) and the version of the domain tree. TreeNodePathId refers to the recorded node in the domain tree. The columns that include "val" are used to store values. ValueUnitEnvId refers to the unit of the value. Comment can store free-text. TreeNodeTime refers to the moment to which the observation applies.

Figure 2 shows an excerpt of the data in the original SDETreenode table in OpenSDE to illustrate how data representation in OpenSDE differs from the conventional approach. In a conventional relational table, each research parameter is represented by one attribute in a table. In OpenSDE, a research parameter is conceptually represented by a node in the domain tree. Actual data related to a research parameter is represented by a row in the OpenSDE data table (SDETreeNode). This table has a fixed number of standard attributes. These attributes are necessary to accommodate the different data types that different research parameters may have. As a result, data representing the value of one research parameter involves only those columns in the row that correspond with its data type.

Querying of row-oriented data is not straightforward. Row-oriented data need to be transformed to a conventional format with a one-column-per-parameter structure to be suitable for analysis with commercially available statistical or graphical programs. Without such transformation queries require self-joins and nested sub queries, which make querying much more complex [11-13].

Entity Export supports the selection of the data to be extracted and export of these data in a conventional format [14]. The user selects the data using the same tree as during data entry. He can also specify which attributes must be exported with each node. Entity Export converts the selected data to a conventional format and exports the resulting tables in a user-specified database.

#### MS Access

MS Access (Microsoft, Redmond) uses a column-oriented relational data model. It is commonly used to build a database, entry forms, and queries for the recording and extraction of data in the context of clinical research projects.

#### Coronary data set

In 1989 the Society of Thoracic Surgeons established a database as an initiative for quality improvement and patient safety.

At the Department of Cardio-Thoracic Surgery (ErasmusMC, Rotterdam, the Netherlands) medical data, related to coronary artery bypass grafting, are currently collected for several research [15] and reporting purposes, and for risk stratification. The data are based on the adult cardiac surgery dataset of the Society of Thoracic Surgeons (STS vs. 2.41) [16]. The STS data set is not exclusively restricted to coronary artery bypass graft (CABG) patient data: other categories like valve or transplantation patients can also be described with this dataset.

The coronary data set is conceptually hierarchical. Data types used are: categorical values, text, integer, date, and real. Complications, for example, can be selected from a list with predefined categories. There are also basic constraints: cause of death can only be entered if date of death is present. A few free text fields were available to accommodate data entry of items not covered in a list.

The data items comprised a total of 173 research parameters where 130 are part of the data collection form of the Adult Cardiac Surgery Database of the Society of Thoracic Surgeons and 46 have been added by the Department of Cardio-Thoracic Surgery.

### Methods

#### Design and application building

OpenSDE: Building a domain model and entry forms.

We defined a domain tree, using the domain model editor of OpenSDE. Data items were ordered in the tree conform their conceptual hierarchy. Properties were assigned to each node to define data type and data entry constraints. Subsequently, we defined several custom entry forms to facilitate data entry. We recorded the time needed for the creation of the tree and the forms.

MS Access: Building a relational database and forms

We first designed a third normal form data model to represent the conceptually hierarchical data set [17]. Subsequently, we created tables, data entry forms, and several constraints, using MS Access.

We called the resulting conventional database application: Thoraxdb.

#### Data entry

Per patient, per procedure, we collected data on preoperative risk factors, operative techniques, postoperative data, and complications.

We entered data of a group of 52 consecutive coronary artery bypass graft (CABG) patients operated at our department of Cardio-Thoracic Surgery in the period June-July 2004 in both the Thoraxdb and OpenSDE data table (SDETreeNode). Paper-based patient records were used as source of patient information.

#### Data extraction

OpenSDE data:

Entity Export was used to generate a conventional relational database from the row modeled data entered with OpenSDE.

All the nodes in the domain tree were chosen for export to conventional column-oriented tables. Subsequently, Entity Export created a copy of the corresponding row-modeled data in a conventional format in MS Access, called EntityExportdb. Unused nodes and attributes were removed during extraction.

MS Access:

Thoraxdb is already in a format suitable for applying SQL queries.

#### Query definitions

The cardio-thoracic surgeons of our hospital defined nine research questions (Table 1) for the purpose of testing the ease of data extraction from the two applications.

**Table 1:** Research questions. Overview of the clinical research questions

	Query name
Q1	How many endarterectomies are performed in 1, 2 and 3 vessel diseased patients
Q2	What is the mean age of patients who underwent only venous revascularization
Q3	What is the number of patients older than 65 years only arterial revascularised as a percentage of the total number of patients.
Q4	Male/Female ratio
Q5	What is the number of complications per operation location.
Q6	What is the number of patients with left main- and 3 vessel disease
Q7	What is the percentage of patients in each hospital with diabetes (Erasmus MC, MCRZ).
Q8	What is the average amount of distal anastomoses per operation.
Q9	What is the number of 1, 2 and 3 vessel diseased patients in Erasmus MC and MCRZ.

The standard facility of MS Access was used for querying both databases (Thoraxdb and EntityExportdb). We translated nine research questions into nine corresponding queries for the Thoraxdb and nine corresponding queries for the EntityExportdb. We compared the query designs, and compared the query results after execution.

## Results

### Design and application building

OpenSDE: Building a domain model and entry forms

Since the original data set was hierarchical by nature, the ordering of the parameters in a domain tree was straightforward. Assigning properties and constraints was also straightforward. Ranges of values and multiple occurrence of nodes were not modeled in the domain tree. The resulting domain tree contained 246 nodes, 66 nodes of which represented context (headers), 7 nodes represented units of measure, and 173 nodes represented the actual research parameters. The difference of 3 parameters with the original data set involves patient ID, gender, and date of birth, which are part of patient registration in OpenSDE.

Building the domain model and 4 custom entry forms (the remaining 13 forms were standard entry forms) took 12 hours.

Thoraxdb: Building a relational database and forms with MS Access

The design of a third normal form data model took 20 hours. Definition of database, entry forms, and macros took 36 hours. The Thoraxdb application contains 300 attributes, divided over 8 tables, and 13 forms.

**Figure 3:**
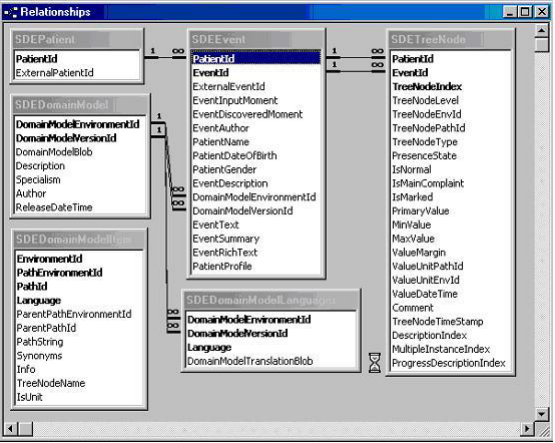
**Screen capture of the table structure of the OpenSDE database**. Schematic representation of the relationships between tables in the OpenSDE database.

**Figure 4:**
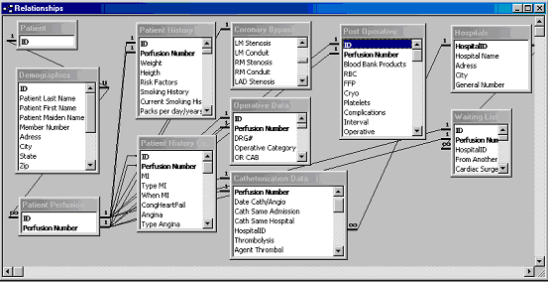
**Screen capture of the table structure of the Thoraxdb**. Schematic representation of the relationships between tables in the Thoraxdb.

The table structures of the OpenSDE database and the Thoraxdb are shown in Figure 3 and 4.

### Data entry

The overall ordering of the data items was very similar as this was determined by the STS data set. Advanced expressiveness of OpenSDE, such as free text comments and progress descriptions were not used. Data entry with OpenSDE was favored over data entry in the Thoraxdb for two main reasons:

- More flexible navigation

- Overview of all entered data

### Data extraction

OpenSDE data

To obtain the conventional equivalent of all data collected with OpenSDE, we used Entity Export to export all content in a conventional format: EntityExportdb. This resulted in 19 tables. Of these two tables are standard: 1 table containing all recording sessions and one table containing data associated with the high levels of the tree. The remaining 17 tables correspond to the 17 main topics in the tree.

Since we exported all data, time using Entity Export was neglectable.

To illustrate the added value of the data conversion by Entity Export, we also designed and executed some queries directly on the SDEtreenode table OpenSDE data format without using Entity Export.

Thoraxdb

Queries were directly designed for the conventional Thoraxdb.

### Query results

Since Thoraxdb and EntityExportdb had a different table structure, there were differences in the design of the nine queries. Differences in query complexity were neglectable. Only query 9 required 1 join in Thoraxdb versus no join in EntityExportdb. The other queries involved either the exact same number of joins and attributes or differed slightly in the number of attributes involved.

All nine queries provided results that corresponded correctly with the content of both databases: exactly all data that met the query criteria (no more and no less) were present in the query results.

## Discussion

### Design

Medical data is by nature hierarchical. In medical descriptions, concepts are described by more detailed concepts. This is also recognizable in the structure of the Table of Contents in medical textbooks. Therefore, creating the domain tree was intuitive and straightforward. To create a good unambiguous domain tree, however, requires thorough understanding of the principles of domain modelling and the conceptual meaning of the various properties. At our hospital clinicians without technical skills actively participate in domain modelling. Ganslandt also reports that such modelling does not require technical skills [18]. Main reason is that a domain model represents metadata in a conceptual format [8]. Knowledge of the domain of application and an analytic mind are needed for domain modelling.

Clinicians cannot be expected to be familiar with normalization: clinicians generally do not receive training in data modelling during their curriculum. Normalization requires both specific training in data modelling and knowledge of the domain of application. Since both skills are rarely present in the same person, the data modeller and the domain expert have to work together. Misunderstanding may result in improper modelling.

An advantage of OpenSDE is the possibility of rapid prototyping. Since OpenSDE generates standard entry forms, based on the domain tree, the modeller can immediately see the effect of any change in the domain tree on data entry.

### Data entry

Although OpenSDE offers more expressiveness (free text comment and progress descriptions are always available), it was not used in the context of this straightforward data set. As mentioned in the results section, the person who performed the data entry in both systems favored OpenSDE for two main reasons:

- Flexibility of navigation: In OpenSDE she could easily navigate to any node in the tree, either by selecting it directly in the tree or via a search string. As data in paper records are not recorded in a fixed order it is convenient to be able to enter the data in the order they become available. In the Thoraxdb, she could only navigate through the forms.

- OpenSDE presents an overview of all data entered during the session in the order of the tree, irrespective of the order or form in which data have been entered. Our data entry person could easily keep track of what she had entered so far. In Thoraxdb, she could only view entered data when she opened the corresponding form.

### Querying

In this study we extracted all data recorded in OpenSDE at once to obtain a conventional equivalent of the Thoraxdb. The number of resulting tables is directly related to the properties assigned to the nodes in the tree. When a node is designated as a 'core entity' all its branching nodes will be exported to one corresponding table. If the modeller assigns the 'core entity' property at a higher level this will result in fewer tables with more attributes.

When using Entity Export in the context of one specific query, the user can make a specific selection of relevant nodes from the tree, which will result in the two standard tables plus as many tables as there are 'core entity' nodes involved.

The use of Entity Export to convert the abstract OpenSDE data format to a conventional table format involves an extra step. The reason for this step is two-fold:

- Data in a conventional format is more easy to query than data in a row-oriented format.

- Since Entity Export presents the domain tree for data selection, the user does not need to select the data set to be analyzed from the less intuitive tables in third normal form.

### Query design

**Figure 5:**
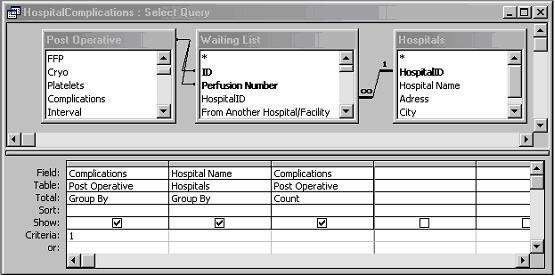
**Design of query 5 in Thoraxdb**. The attribute Complications represents if a complication occurred or not, and attribute Hospital Name refers to the place of operation.

**Figure 6:**
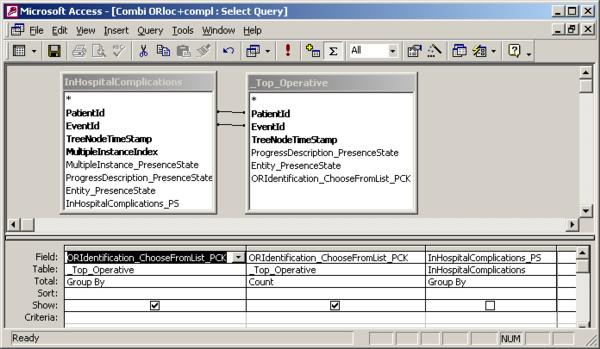
**Design of query5 in EntityExportdb**. Attribute ORIdentification_ChooseFromList_PCK represents the place of operation. Attribute InHospitalComplications_PS represents if a complication occurred or not.

**Figure 7:**
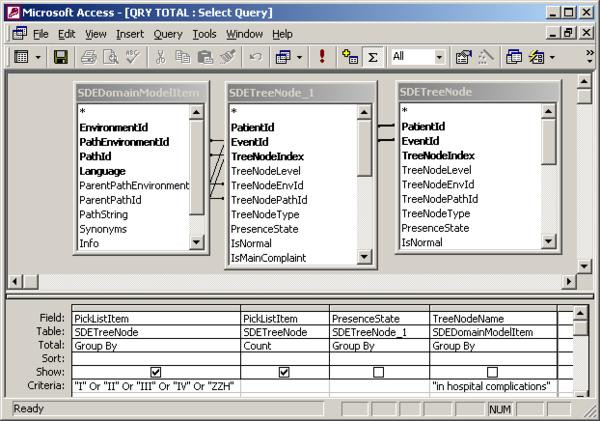
**Design of query5 in the original SDETreeNode table**. Attribute PickListItem represents a categorical value. This query filters for values representing surgery rooms. Attribute TreeNodeName in table SDEDomainModelItem contains the name of a node, and is needed to filter SDETreeNode for "in hospital complications". Attribute PresenceState represents whether a node has been entered as present or absent. The inner join of table SDETreeNode is needed to combine the proper rows (such that "in hospital complications" and surgery room value were part of the same registration).

To illustrate the query differences between the two conventional databases we show the design of Query 5 (What is the number of complications per operation location?) for the conventional Thoraxdb (figure 5) and the EntityExportdb (figure 6). Although it is possible to query the row-oriented SDETreeNode table directly, this requires more effort. To illustrate this, we also include the design of Query 5 for the original SDETreeNode (prior to using Entity Export) table in this example (figure 7).

Design of Q5 was most complex in the original SDETreeNode table (1 step, 3 attributes and 2 joins), and execution took noticeably more time than query 5 in the other 2 databases. This is a result of the abstraction in the original SDETreeNode table. First, a specific parameter (treenode) can only be extracted via the link between the TreeNodePathId in the data table (SDETreeNode) and the TreeNodeName in a reference table (SDEDomainModelItem). Second, a query involving two research parameters involves 1 inner join of the data table SDETreeNode. SDETreeNode has to be joined with itself (inner join) because the data representing place of operation and complications are stored in different rows of the same table. Hence, three different parameters would require 2 inner joins. In fact, n parameters require n-1 inner joins. Inner joins exponentionally slow down query execution. Direct querying of the original SDETreeNode table is, therefore, only feasible for relatively simple queries, involving 1 or two attributes. Entity Export solves this problem through data conversion from the abstract row-oriented structure to conventional tables.

It should be mentioned, that the result for one of the queries in the Thoraxdb could only be obtained after specific data manipulation. Three fields (related to the number and type of distal anastomoses) had categorical values: none, 1,2,3,4, or 5. We converted the data type to integer and the values to 0 through 5 respectively. The numerical representation was required for the calculation of an average amount of distal anastomoses per patient.

As illustrated by the need to change categorical values into numeric values, it is important at design time to take into account the type of research questions that will need to be answered. However, one can never foresee all research questions in advance and different research questions may involve different design preferences.

Apart from the definition of data types, relationships between tables, or the ordering of concepts in a domain tree, one has to be cautious with respect to semantic equivalence: the possibility to enter the same finding in more than one way. Semantic equivalence is undesirable, irrespective of any research question, as it increases the chance of inconsistent data or overlooking data. Hence, thorough study of the semantics of the data model is essential prior to querying.

### Recording data for research and patient care

In research settings, recording of the research data items usually happens in addition to data collection for routine care. To put the research data in the proper context, part of the data needs to be recorded twice.

Ideally, data recording for research is integrated with data recording for routine patient care. Using a conventional data model for structured data recording in the context of patient care has been extremely challenging, especially for large medical domains. The main reason is that the data set that needs to be entered is unpredictable: very many findings are possible while a few apply to a specific patient.

OpenSDE accommodates data recording for research as well as routine care, also in large domains like general pediatrics [4]. It is important to realize that the results of our example with the coronary data set apply to a very well-defined data set for prospective research. The recording of this data set involved the same expressiveness as was accommodated in the Thoraxdb, which is only a small part of the expressiveness that OpenSDE offers.

Analysis of routinely recorded patient data shows that users actually use the expressiveness of OpenSDE as they prefer. The large expressiveness of OpenSDE implies freedom of choice how to record data, which results in differences in recording of the same findings. Physicians appear to differ in their use of free text, they differ in level of detail with which they record, but they may also choose different concepts during data entry[14]. The latter is related to how users map their findings to the concepts in the domain tree. When a routinely used term is not present as such in the tree, the user must map the term to one of the concepts in the tree. Such mappings explain part of the differences. Also, some users may enter that auscultation of the lungs is 'normal' while others enter 'vesicular breath sounds'.

An important pitfall in modelling is semantic redundancy, which is present when a concept is represented by more than one node or combination of nodes in the tree. Semantic redundancy may also cause differences in data recording. When clinicians are new to modelling, they tend to model the context in which they address medical topics. For example, 'edema' is relevant in the context of cardiac disease, renal disease, trauma, infection, etc. Each concept, however, has to be represented once in a domain tree, whereas it may occur on multiple forms. The differences found in routinely recorded data make querying difficult and complex. Hurdles in data extraction are free text, missing data, and conceptual identical data that are structured differently [6].

The results of the current study indicate that well-defined research data sets that can be accommodated with a conventional database can also be accommodated with OpenSDE with comparable query effort. For such datasets, however, OpenSDE presents a more intuitive and flexible strategy for the construction of database and user interface. OpenSDE offers conditional data checklists to promote the completeness and unambiguity required for that subset of care data, needed for research.

### Differences between OpenSDE and the conventional approach

Relational databases are pre-eminently suited for businesses, who record which customers order which products, which suppliers offer which parts, the progress of orders, amounts in stock, etc.[17]. These data are characterized by the fact that they are highly related. In such cases, the relational model corresponds well to how that domain is perceived. When data are descriptive and by nature hierarchical, a domain tree is far more intuitive.

When flexibility in content is important, i.e. when one expects the required content to change relatively frequently over time, a row-model is the preferred choice.

Another issue pertains to the required level of customization of the application. OpenSDE has many features for customization without the need for programming [2]. These features are comparable to wizard functionality and form properties in MS Access. When more specific customization is needed both OpenSDE and MS Access will require program code.

In the next version of OpenSDE, currently under development, expressiveness is more explicitly modelled, and unpredictable use of expressiveness by the user can be controlled as needed.

## Conclusion

In this study, OpenSDE was experienced as an flexible and intuitive tool to create a database and data entry interface for the collection of data in the context of well-defined prospective clinical research projects.

Entity Export provided an intuitive way to select data for analysis. Although query reliability and complexity differ slightly between Entity Export generated output and conventional databases, query effort is comparable.

An important pitfall, that is independent of the choice of a row model or conventional model, is the danger of semantic redundancy. This danger requires specific attention while designing and querying databases. Minimizing options for unpredictable, ad hoc, data entry will greatly help to improve the analyzability of the data set.

OpenSDE offers intuitive design and the flexibility to cope with quickly changing and evolving datasets, whereas Entity Export provides an adequate tool to produce data in a conventional format. For prospective studies involving well-defined and straightforward data sets, OpenSDE deserves to be considered as an alternative to the conventional approach.

## Competing interests

The authors declare that they have no competing interests.

## Authors' contributions

ACV had primary responsibility for design and conception of the study, participated in analysis of the data, writing and revising of the manuscript.

AMvG participated in the conception and design of the study, analysis of the data and has been involved in revising the manuscript.

MdW participated in interpretation of the data and has been involved in revising the manuscript.

AB participated in the conception of the study and the revising of the manuscript.

All authors read and approved the final manuscript.

## Pre-publication history

The pre-publication history for this paper can be accessed here:


